# Biofilm-Grown *Burkholderia cepacia* Complex Cells Survive Antibiotic Treatment by Avoiding Production of Reactive Oxygen Species

**DOI:** 10.1371/journal.pone.0058943

**Published:** 2013-03-13

**Authors:** Heleen Van Acker, Andrea Sass, Silvia Bazzini, Karen De Roy, Claudia Udine, Thomas Messiaen, Giovanna Riccardi, Nico Boon, Hans J. Nelis, Eshwar Mahenthiralingam, Tom Coenye

**Affiliations:** 1 Laboratory of Pharmaceutical Microbiology, Ghent University, Gent, Belgium; 2 Dipartimento di Biologia e Biotecnologie “Lazzaro Spallanzani”, Università degli Studi di Pavia, Pavia, Italy; 3 Organisms and Environment Division Cardiff School of Biosciences, Cardiff University, Cardiff, United Kingdom; 4 Laboratory of Microbial Ecology and Technology (LabMET), Ghent University, Gent, Belgium; Vrije Universiteit Brussel, Belgium

## Abstract

The presence of persister cells has been proposed as a factor in biofilm resilience. In the present study we investigated whether persister cells are present in *Burkholderia cepacia* complex (*Bcc*) biofilms, what the molecular basis of antimicrobial tolerance in *Bcc* persisters is, and how persisters can be eradicated from *Bcc* biofilms. After treatment of *Bcc* biofilms with high concentrations of various antibiotics often a small subpopulation survived. To investigate the molecular mechanism of tolerance in this subpopulation, *Burkholderia cenocepacia* biofilms were treated with 1024 µg/ml of tobramycin. Using ROS-specific staining and flow cytometry, we showed that tobramycin increased ROS production in treated sessile cells. However, approximately 0.1% of all sessile cells survived the treatment. A transcriptome analysis showed that several genes from the tricarboxylic acid cycle and genes involved in the electron transport chain were downregulated. In contrast, genes from the glyoxylate shunt were upregulated. These data indicate that protection against ROS is important for the survival of persisters. To confirm this, we determined the number of persisters in biofilms formed by catalase mutants. The persister fraction in Δ*katA* and Δ*katB* biofilms was significantly reduced, confirming the role of ROS detoxification in persister survival. Pretreatment of *B. cenocepacia* biofilms with itaconate, an inhibitor of isocitrate lyase (ICL), the first enzyme in the glyoxylate shunt, reduced the persister fraction approx. 10-fold when the biofilms were subsequently treated with tobramycin. In conclusion, most *Bcc* biofilms contain a significant fraction of persisters that survive treatment with high doses of tobramycin. The surviving persister cells downregulate the TCA cycle to avoid production of ROS and at the same time activate an alternative pathway, the glyoxylate shunt. This pathway may present a novel target for combination therapy.

## Introduction

*Burkholderia cenocepacia* is a member of the *Burkholderia cepacia* complex (*Bcc*), a group of 17 closely related and phenotypically similar species [1]. These bacteria are opportunistic pathogens that can cause severe lung infections in immunocompromised people, including cystic fibrosis (CF) patients [2]. The prevalence and outcome of infections appear to be species dependent and infections with *Burkholderia multivorans* and *B. cenocepacia* are associated with high rates of transmission and high mortality [3]. *B. cenocepacia* J2315 belongs to the highly transmissible ET12 lineage which has infected many CF patients in Canada, the UK and various European countries [4]. Unfortunately, *Bcc* organisms are difficult to eradicate because of their innate resistance to a wide range of antibiotics and their capacity to form biofilms [5]. Mechanisms of resistance include changes in lipopolysaccharide structure, the presence of several multidrug efflux pumps, inducible chromosomal β-lactamases and altered penicillin-binding proteins [6]. In addition, biofilm-associated cells are often more tolerant to antimicrobials than planktonic cells [7] due to reduced drug penetration in the biofilm, the lower growth rate of sessile cells and/or the expression of specific resistance genes [8], [9]. Even when patients are treated with high doses of antibiotics known to be effective against *Bcc* species *in vitro,* it is often impossible to clear the infection [10].

Persister cells, cells that have entered a dormant multidrug-tolerant state, are thought to be involved in the recalcitrance of biofilm related infections [11]. These persisters are not mutants but phenotypic variants of the wild type [11]. Upon treatment of a biofilm with high doses of an antibiotic most of the bacteria are killed, but some of them neither grow nor die. After removal of the antibiotic, these surviving cells will start to grow and will give rise to a new infection. The resulting biofilm will again only contain a very small fraction of persister cells [12]. The mechanisms leading to persistence are not well understood. Screening knockout mutant libraries has not led to the identification of mutants completely lacking persister cell formation, suggesting that dormancy mechanisms are redundant [11]. Currently, it is assumed that toxin/antitoxin modules are involved in persister formation [12]. Toxins are proteins that inhibit important cellular functions such as translation or replication. This condition can be reversed by the expression of the corresponding antitoxin which can form an inactive complex with the toxin. By causing a reversible dormant state, toxins protect bacteria against antibiotics which require active targets in order to be effective [11]. The phenomenon of persister formation in exponentially growing planktonic cultures has been demonstrated for several micro-organisms (including *Escherichia coli, Pseudomonas aeruginosa, Staphylococcus aureus* and *Mycobacterium tuberculosis*) [11], but has so far not been investigated in the *Bcc*.

Recently, Kohanski et al. demonstrated that the production of reactive oxygen species (ROS) contributes to the antimicrobial activity of bactericidal antibiotics. The primary drug-target interactions stimulate the oxidation of NADH via the electron transport chain, which itself is dependent on the tricarboxylic acid cycle (TCA). A hyperactivation of the electron transport chain results in increased superoxide formation, leading to damage to iron-sulfur clusters in proteins with the release of ferrous iron. This ferrous iron can be oxidised in the Fenton reaction with the production of hydroxyl radicals capable of damaging proteins, DNA and lipids and ultimately leading to cell death [13].

The glyoxylate cycle is an anaplerotic pathway of the TCA cycle, which bypasses the decarboxylation steps in which NADH is produced. This pathway allows microorganisms to utilize simple carbon compounds as a carbon source [14]. The glyoxylate cycle is absent in humans, making it an interesting drug target [14]. For example, Van Schaik et al [15] found that inhibition of isocitrate lyase (ICL, the first enzyme of this shunt), by itaconate during experimental chronic *Burkholderia pseudomallei* lung infections forces the infection into an acute state, which can then be treated with antibiotics.

In the present study we wanted to investigate whether persister cells are present in *Bcc* biofilms, what the molecular basis of antimicrobial tolerance in *Bcc* persisters is, and how persisters can be eradicated from *Bcc* biofilms.

## Materials and Methods

### Strains and Culture Conditions

The strains used in the present study are shown in Tables 1 and 2 [16], [17]. All strains were cultured at 37°C on Luria-Bertani agar (LBA, Oxoid, Hampshire, UK) or on Mueller Hinton agar (MHA, Oxoid). Overnight cultures were diluted in Luria-Bertani broth (LBB, Oxoid) and incubated aerobically at 37°C. The succinate dehydrogenase antisense overexpression mutants were grown on LBA supplemented with 800 µg/ml trimethoprim (Tp) (Ludeco, Brussels, Belgium) or in LBB supplemented with 800 µg/ml Tp and 0.2% rhamnose (Sigma Aldrich, Bornem, Belgium).

**Table 1:** Strains used in the present study.

Strain	LMG number	Strain information	Source (reference)
*B. cenocepacia* J2315	LMG 16656	CF patient, UK, ET12 strain	BCCM/LMG Bacteria Collection
*B. cenocepacia* K56-2	LMG 18863	CF patient, Canada	BCCM/LMG Bacteria Collection
*B. cenocepacia* C5424	LMG 18827	CF patient, Canada	Miguel Valvano [16]
*B. cenocepacia* MDL1		C5424 Δ*katA* mutant strain	Miguel Valvano [16]
*B. cenocepacia* MDL2		C5424 Δ*katB* mutant strain	Miguel Valvano [16]
*B. cenocepacia* D2		ΔBCAL2118ΔBCAM1588	This study
*B. cenocepacia* SDH7		Overexpression of BCAM0967	This study
*B. cenocepacia* SDH8		Overexpression of BCAM0968	This study
*B. ambifaria* AMMD	LMG 19182	Pea rhizosphere, USA	BCCM/LMG Bacteria Collection
*B. cenocepacia* HI2424	LMG 24507	Soil, USA, PHDC strain	John LiPuma [17]
*B. lata* ATCC 17769	LMG 6992	Soil, Trinidad and Tobago	BCCM/LMG Bacteria Collection
*B. multivorans* ATCC 17616	LMG 17588	Soil, USA	BCCM/LMG Bacteria Collection
*B. vietnamiensis* ATCC 53617	LMG 22486	Wastewater, USA	BCCM/LMG Bacteria Collection
*B. dolosa* R-5670	LMG 18943	CF patient, US	BCCM/LMG Bacteria Collection
*B. contaminans* CCUG 34411	LMG 16227	CF patient, Sweden	BCCM/LMG Bacteria Collection
*B. ubonensis* NCTC 13147	LMG 20358	Surface soil, Thailand	BCCM/LMG Bacteria Collection
*B. cepacia* ATCC 25416	LMG 1222	*Allium cepa*, US	BCCM/LMG Bacteria Collection
*B. pyrrocinia* ATCC 15958	LMG 14191	Soil, US	BCCM/LMG Bacteria Collection

**Table 2:** The MIC and fraction of persisters for early and late *Bcc* clonal isolates obtained from infected CF patients.

Species	Strain	Time interval (years)		MIC	Average % persisters(n = 3)	SD
**Clonal isolates from Canada**						
*B. multivorans*	C8298	8	Early	64	0.1551	0.2352
	D2156		Late	64	0.0068	0.0076
*B. multivorans*	C8814	5	Early	32	0.3411	0.3080
	D0999		Late	128	0.0012	0.0008
*B. multivorans*	C6396	9	Early	64	0.0031	0.0004
	D0913		Late	128	0.0005	0.0003
*B. cenocepacia*	C4053	6	Early	128	0.0004	0.0004
	C6121		Late	64	0.0019	0.0009
*B. cenocepacia*	C3921	10	Early	1024	0.0249	0.0270
	C9343		Late	1024	0.0051	0.0044
*B. cenocepacia*	C6483	4	Early	256	0.0019	0.0031
	C8474		Late	256	0.0214	0.0363
*B. cenocepacia*	C4629	7	Early	128	0.0226	0.0326
	C8482		Late	128	0.0036	0.0007
*B. cenocepacia*	C5876	8	Early	128	0.0020	0.0004
	D0465		Late	256	0.0000	0.0001
*B. cenocepacia*	C5424	3	Early	256	0.0527	0.0753
	C7376		Late	128	0.0114	0.0152
**Clonal isolates from the US**						
*B. cenocepacia*	AU0326	11	Early	256	0.0066	0.0095
	AU18962		Late	512	0.0276	0.0441
*B. cenocepacia*	AU0734	13	Early	256	0.0010	0.0007
	AU21801		Late	256	0.0001	0.0001
*B. vietnamiensis*	AU0808	12	Early	16	0.0019	0.0025
	AU21645		Late	8	0.0092	0.0116
*B. dolosa*	AU3503	10	Early	32	0.0040	0.0044
	AU21993		Late	32	0.0821	0.0702
*B. dolosa*	AU0265	14	Early	128	0.9246	0.7618
	AU21961		Late	128	0.1230	0.1847

### Minimal Inhibitory Concentration (MIC)

MICs were determined in duplicate according to the EUCAST broth microdilution protocol using flat-bottom 96-well microtiter plates (TPP, Trasadingen, Switzerland) [18]. Tobramycin and ciprofloxacin (Sigma Aldrich) concentrations tested ranged from 0.25 to 1024 µg/ml and from 0.25 to 128 µg/ml, respectively. Itaconate and 3-nitropropionate (3-NP) (Sigma Aldrich) concentrations ranged from 0.20 to 100 mM. 2-Thenoyltrifluoroacetone (Sigma Aldrich) concentrations ranged from 5 to 625 nM. The MIC was defined as the lowest concentration for which no significant difference in optical density (λ = 590 nm) was observed between the inoculated and blank wells after 24 h incubation. Differences in absorbance were considered significant when the 95%-confidence interval (calculated using Microsoft Excel) did not contain zero. All MIC determinations were performed in duplicate and replicates never differed more than two fold. When a two fold difference was observed between replicates, the lowest concentration was recorded as the MIC.

### Quantification of Persister Cells in Biofilms and Planktonic Cultures

To determine the number of surviving cells, 24 h old biofilms or planktonic cultures were exposed to tobramycin, an aminoglycoside antibiotic, or ciprofloxacin, a fluoroquinolone, in concentrations ranging from 0.5 to 64× the MIC for 24 h. Biofilms were grown in the wells of a round-bottom 96-well microtiter plate (TPP), as described previously [19]. After 24 h of growth, the supernatant was removed and 120 µl of an antibiotic solution in physiological saline (PS) or 120 µl PS ( = control) was added and the plates were further incubated at 37°C. Twelve wells were used for each condition. After 24 h of treatment, cells were harvested by vortexing and sonication (2×5 min) (Branson 3510, Branson Ultrasonics Corp, Danbury, CT) and quantified by plating on LB (n ≥2 for all experiments). For the planktonic experiments an overnight culture was diluted to an optical density of 0.1 (approximately 10^8^ cells/ml). After an additional 24 h growth in a shaking warm water bath, cell suspensions with an optical density of 1 (approximately 10^9^ cells/ml) were transferred to falcon tubes and centrifuged for 9 min at 5000 rpm. Cells were resuspended in an antibiotic solution in PS, or in PS and further incubated at 37°C. After 24 h of treatment the tubes were centrifuged, resuspended in PS and quantified by plating on LB (n ≥2 for all experiments).

### RNA Extraction and Microarray Analysis

Biofilms were grown as described above and exposed to an antibiotic (concentration of 4×MIC) or a 0.9% NaCl solution (untreated controls) for 24 h. Treated and untreated *B. cenocepacia* J2315 sessile cells were harvested by vortexing (5 min) and sonication (5 min) and transferred to sterile tubes. RNA was extracted immediately following harvesting of the cells, using the Ambion RiboPure Bacteria Kit (Ambion, Austin, TX) according to the manufacturer’s instructions and the procedure included a DNase I treatment for 1 h at 37°C. After extraction, RNA of both treated and untreated samples was concentrated using Microcon YM-50 filter devices (Milipore, Billerica, MA) and linearly amplified using the MessageAmp II-Bacteria Kit (Ambion) prior to the microarray analysis. The method was first optimised with the use of standard *Escherichia coli* RNA. Assessment of RNA yield and quality, cDNA synthesis and the hybridization and washing of the custom made 4×44K arrays (Agilent, Santa Clara, CA) was performed as described previously [20]. The gene expression analysis was performed using GeneSpring GX 7.3 (Agilent) and data were normalized using the procedure recommended for two-colour Agilent microarrays. Only features dedicated to *B. cenocepacia* J2315 that were labelled “present” or “marginal” were included in the analysis. After this initial filtering, a student’s T-test analysis was performed (p<0.05). The experimental protocols and the raw microarray data can be found in ArrayExpress under the accession number E-MEXP-3532.

### Quantitative RT-PCR

In order to validate the microarray results, the expression of 11 selected genes was examined using RT-qPCR. Biofilms were treated and RNA was extracted as described above. cDNA was synthesized using the iScript cDNA Synthesis Kit (Bio-Rad, Hercules, CA). Forward and reverse primers were developed using tools available on the NCBI website and they were compared with the *B. cenocepacia* J2315 genome sequence using BLAST to determine their specificity (Table 3). The primer concentration used was 600 nM (300 nM for BCAM1588). All qPCR experiments were performed on a Bio-Rad CFX96 Real-Time System C1000 Thermal Cycler as described previously [20]. Each sample was spotted in duplicate and control samples without added cDNA were included in each experiment. The initial 3 min denaturation step at 95°C was followed by 40 amplification cycles, consisting of 15 s at 95°C and 60 s at 60°C. A melting curve analysis was included at the end of each run. To allow accurate normalization of our data, we also included three reference genes (BCAL2694, BCAS0175, BCAM2784) for which we confirmed expression stability using GeNorm [21] prior to the actual analyses (Figure S1).

**Table 3:** Primer sequences.

Gene	Annotation	FW primer	RV primer
**Glyoxylate shunt**			
BCAL2122	Malate synthase	GGCCGACCGCTCGAAGATCA	CGCCTTGTCGTTCGCTTCCG
BCAL2118	Isocitrate lyase	GCACCGTACGCCGACCTGAT	GCTTGCCCGGGAACTGCTTG
BCAM1588	Isocitrate lyase	TCCTTGCCCGCTGCCTTCAT	ATCCCGAACGCGAAGCTGGT
**Tricarboxylic acid cycle**			
BCAM0961	Aconitate hydratase	CTCGGCCAGCCGGTGTACTT	CAGCGACTTCGTGCCTTCGC
BCAM2701	Aconitate hydratase	CGGTGATCCAGGCGCAGGAT	CAGGCGCATCCACGATGCAC
BCAM1833	Aconitate hydratase/methylisocitrate dehydratase	GCATGCTGACGGTCGCCAAG	GGTCGGGCAGGCCTTCGATT
BCAL2735	Isocitrate dehydrogenase	CGCTGAAGCCGGAAGCGAAC	ACCGCGCTGCCCTTGATCTT
BCAL2736	Isocitrate dehydrogenase	ATCAAGGGGCCGCTCACGAC	GGCGCAGGCACACGTAGAGA
BCAL1517	Dihydrolipoamide dehydrogenase	GAAGTGAGCGGCGAGGGTGA	TCGGCACCGAGTCGAACGTC
BCAL0957	Succinyl-CoA synthethase subunit alpha	ACGCAGGGCATTACCGGCAA	CGCGGCCGTTTGCGTATTCA
BCAM0970	Succinate dehydrogenase iron-sulfur subunit	CCTCCAGTCGCCGGAAGAGC	CACCAGAAGCTCGGGCACGA
BCAM0965	Malate dehydrogenase	CCGGTCGCGTCGATCGAGAA	GCGAAGCGGAAGTCCGGGTA
**Other genes**			
BCAM2318	Putative ferredoxin oxidoreductase	TGAAGCTGATCGCGCCGGAT	GCGCACCGCCTTCATGTACG
**Reference genes**			
BCAL0036	ATP synthase beta chain	CAAGACCGTCAACATGATGGA	TCGAGTCCTTCATTTCGTGGTA
BCAL0289	Glutamate synthase	ATCATCCAGCAGGGTCTGAAGA	GCCATTTCCTCGCGATAGAA
BCAL0421	DNA gyrase B subunit	GTTCCACTGCATCGCGACTT	GGGCTTCGTCGAATTCATCA
BCAL1459	Calcineurin-like phosphoesterase	ATCCCTTGAAATCGAGCATCA	TACGCTCGACAACGTGCATTA
BCAL1659	Ribose transport permease	ACCGTGTTCGGACGCTATCT	CATCAGTGCGAATACGAGAATTTT
BCAL1861	Acetoacetyl-CoA reductase	GATCACCTGCTTCGTGACGTT	GACGTCGTGTTCCGCAAGAT
BCAL2694	Dehydrogenase	CTTGCCGTGATCCTCGAGAT	GAGATCAGCGAGGCCGAGTA
BCAM0991	Tryptophane synthase beta chain	GCCAACGTCTACCGGATGAA	GACCGTGCCGATGATGTAGAA
BCAM2784	Aminotransferase	CCCCGTTCTCGCTCTACGT	GTGTCGCCGAGGCAGAAAT
BCAS0175	Hydrolase	ATGGCCAGTTCGCTCATCA	ACGCGATGTCGATACTCGAAT

### Flow Cytometry

The induction of ROS by tobramycin was confirmed by staining of treated (4×MIC tobramycin, 24 h) and untreated biofilms with the ROS-specific fluorescent dye 2′,7′-dichlorodihydro-fluorescein diacetate (DCFHDA, Sigma Aldrich), followed by quantification of labeled cells by flow cytometry. After 24 h of treatment, as described above, 22 µl DCFHDA (0.1 mM) was added to each well. After a 30 min incubation at 37°C in the dark, the wells were rinsed and 100 µl PS was added. Tobramycin-treated and untreated sessile cells were harvested by scraping and then transferred to sterile tubes. The tubes were centrifuged (for 6 min, at 9000 rpm), the cells were resuspended and 100 fold diluted in PS. All solutions were prepared using MQ water (Millipore, Billerica, MA) and were filter-sterilized before use (Puradisk FP30; Whatman, Middlesex, UK). Labeled cells were quantified using a Cyan ADP flow cytometer (Beckman Coulter, Suarlée, Belgium) with a 488 nm argon laser and a 530–540 nm emission filter. Data of at least 50000 cells were collected for each sample.

### Effect of Superoxide Dismutase (SOD), Isocitrate Lyase (ICL) or Succinate Dehydrogenase (SDH) Inhibition on Survival of *Bcc* Persisters

To confirm the importance of protection against ROS for the survival of persister cells, the number of surviving cells was quantified in biofilms treated with tobramycin (4×MIC, 24 h) in combination with the SOD inhibitor diethyldithiocarbamate (0.05 mM) (Sigma Aldrich). To confirm the importance of ICL for persistence, the number of surviving cells was quantified in treated (4×MIC, 24 h) and untreated biofilms grown in LBB supplemented with 50 mM itaconate or 10 mM 3-NP, two ICL inhibitors. These concentrations were below the MIC of these inhibitors for the strains tested (data not shown). Itaconate and 3-NP solutions were neutralised with NaOH in LBB and sterilized by filtration. To evaluate the role of SDH in persistence, the number of surviving cells was determined in biofilms grown in LBB supplemented with 250 nM 2-thenoyltrifluoroacetone, a concentration well below the MIC (data not shown).

### Construction of ICL Mutant

The genome of *B. cenocepacia* J2315 contains two ICL encoding genes, BCAL2118 and BCAM1588. To confirm their importance, we constructed a double ICL mutant. All *B. cenocepacia* mutant strains were constructed following the protocol described by Hamad et al., which allows for the creation of unmarked nonpolar gene deletions [22]. Briefly, this mutagenesis procedure requires the upstream and downstream regions flanking the target gene to be cloned into pGPI-*Sce*I-XCm plasmid. The PCR amplifications of these regions (about 500 bp each) were performed with the HotStar HiFidelity polymerase kit (QIAGEN, Milan, Italy) according to the manufacturer’s instructions. The following primer pairs were used for the deletion of BCAL2118 (*aceA*): KOaceXL (5′-TTTCTAGAATTTCACGATACGGGA-3′ *Xba*I site is underlined), KOaceBL (5′-TTGGATCCCGGACAGGTAGATGGC-3′ *Bam*HI site is underlined), KOaceBR (5′-TTGGATCCACAAGGGCTTCACGGC-3′ *Bam*HI site is underlined), and KOaceKR (5′-TTGGTACCCTATCTCGGCATCATC-3′ *Kpn*I site is underlined). The following primer pairs were used for the deletion of BCAM1588: KO1588XL (5′-TTTCTAGATGACCGATCCGCACGC-3′ *Xba*I site is underlined), KO1588BL (5′-TTGGATCCCGACCGACAACCTGGC-3′ *Bam*HI site is underlined), KO1588BR (5′-TTGGATCCCTTGTTCTGGGCACGC-3′ *Bam*HI site is underlined), and KO1588KR (5′-TTGGTACCATCAAAGAATTGTCC-3′ *Kpn*I site is underlined). The mutagenesis plasmids were conjugated into *B. cenocepacia* J2315 and also into the ΔBCAL2118 mutant strain, in order to create the ΔBCAL2118ΔBCAM1588 double mutant (designated as D2). Exconjugants were selected in the presence of Tp (200 µg/ml), chloramphenicol (400 µg/ml)and ampicillin (200 µg/ml). The insertion of the mutagenesis plasmid into *B. cenocepacia* genome was confirmed by spraying catechol on the cells, which turn yellow in the presence of this compound and 2,3-catechol-dioxygenase, encoded by *xylE* (located in pGPI-*Sce*I-XCm plasmid). Subsequently, pDAI-*Sce*I-*Sac*B plasmid (encoding the I-SceI endonuclease), was mobilized by conjugation. Site-specific double-strand breaks take place in the chromosome at the I-SceI recognition site, which is present in pGPI-*Sce*I-XCm plasmid, resulting in tetracycline-resistant (due to the presence of pDAI-*Sce*I-*Sac*B) and Tp-susceptible (indicating the loss of the integrated plasmid) exconjugants. The gene deletions were confirmed by PCR reaction and sequencing of the amplification products. The isolation of deletion mutants cured from pDAI-*Sce*I-*Sac*B was achieved by growing *B. cenocepacia* cells on LB medium without antibiotics and then screening the resulting colonies for loss of tetracycline resistance.

### Construction of SDH Antisense Overexpression Mutants

To confirm the importance of SDH we constructed an antisense overexpression mutant. Succinate dehydrogenase contains 4 subunits. Gene encoding for the two hydrophobic subunits *sdhC* and *sdhD* (BCAM0967 and BCAM0968) were amplified by PCR using a Phusion^R^ high Fidelity PCR Kit (Bioké NEB, Leiden, the Nederlands). The following primers were used for amplifying BCAM0967: forward primer GTACAAGCATATGTTAGAATGCTCCGAACA (*Nde*I restriction site is underlined) and reverse primer ATTCTAGAATGACTGACGCAGTAAGAAAGC (*Xba*I restriction site is underlined). The following primers were used for amplifying BCAM0968: forward primer GTACAAGCATATGTTACACTCTCCAGAGAA (*Nde*I restriction site is underlined) and reverse primer AATCTAGAATGGCAGCCAACAACCGAATC (*Xba*I restrictions site is underlined). Cycling conditions were 30s 98°C, 30 cycli of 10s 98°C, 30s 60°C, and 24s 72°C, and 10 min 72°C. PCR products were purified with a High Pure PCR product purification kit (Roche, Vilvoorde, Belgium), digested and subsequently ligated into a plasmid described by Cardona et al. [23], with a rhamnose-inducible promotor and a Tp selection marker. DNA ligations, restriction endonuclease digestions, and agarose gel electrophoresis were performed according to standard techniques [24]. Restriction enzymes and T4 DNA ligase were purchased from Bioké NEB. Plasmid transformation experiments with *E. coli* DH5α were carried out by the calcium chloride method [25]. Resistant colonies were isolated and screened for the presence of the construct. Plasmids were transferred into *B. cenocepacia* by triparental mating [26] using pRK2013 as a helper plasmid [27]. Exconjugants were selected on LB agar plates supplemented with 800 µg/ml Tp and 50 µg/ml gentamicin.

## Results and Discussion

### Presence of Persister Cells

The presence of persister cells has been proposed as an important factor in biofilm resilience [8]. To investigate the presence of these dormant, multidrug-tolerant phenotypic variants in *Bcc* biofilms, mature biofilms were treated with tobramycin, an aminoglycoside antibiotic, frequently used in the treatment of infected CF patients. The experiments were carried out using *B. cenocepacia* J2315, a member of the epidemic ET12 lineage of which the genome has been sequenced. The MIC was previously determined to be 256 µg/ml [18]. Biofilms were treated with various concentrations of tobramycin, ranging from 0.5 to 64×MIC for 24 h.

**Figure 1:**
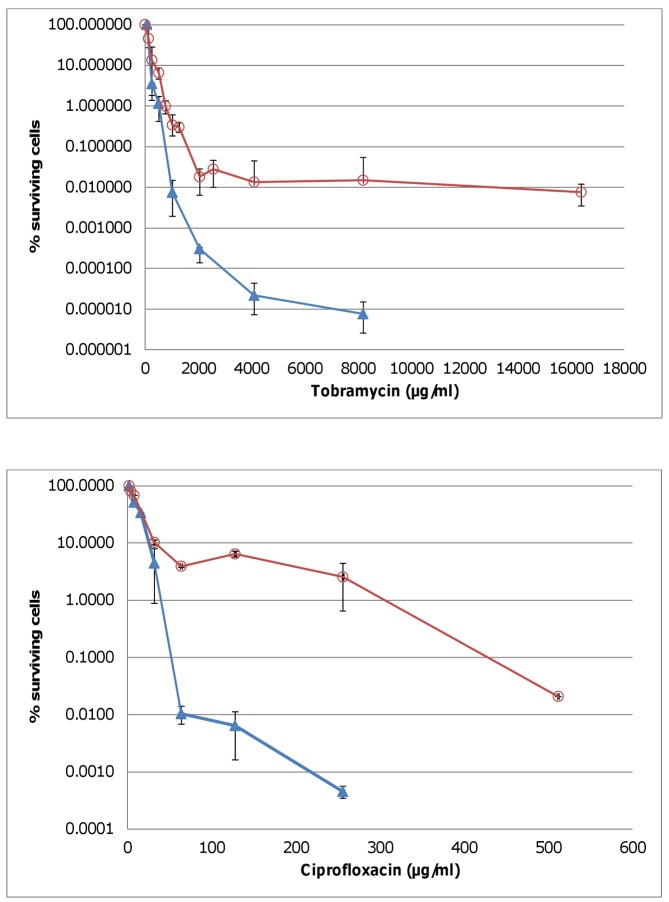
The percentage surviving cells after treatment of *B. cenocepacia* J2315 with tobramycin or ciprofloxacin. The results from the biofilm experiments are indicated with open circles and those from the planktonic cultures with triangles. Error bars represent min-max (n ≥2).

We found that most cells were killed when J2315 biofilms were treated with tobramycin in concentrations ranging from 1 to 4×MIC, but even at higher concentrations (up to 64×MIC), some of the cells survived, indicating the presence of persisters (Fig. 1). Similar results were obtained for other *Bcc* strains (Table 4). Treatment with ciprofloxacin, a fluoroquinolone, resulted in an even higher fraction of surviving cells (Fig. 1). The presence of persisters is not unique to biofilms as we found similarly-shaped survival curves in planktonic cultures, although the fraction of surviving cells was considerably lower (Fig. 1).

**Table 4:** The average percentage surviving cells for different *Bcc* strains after treatment of biofilms with high concentration of tobramycin or ciprofloxacin (n ≥3).

	Tobramycin (4×MIC)		Ciprofloxacin (4×MIC)	
Strain	% surviving cells	SD	% surviving cells	SD
*B. cenocepacia* J2315	0.3409	0.1877	10.2425	1.4721
*B. cenocepacia* K56-2	0.0064	0.0072	3.7081	3.5486
*B. cenocepacia* C5424	0.0319	0.0038	37.9861	15.6789
*B. cenocepacia* HI2424	0.0007	0.0007	3.6601	0.6902
*B. ambifaria* AMMD	0.0096	0.01424	14.6604	11.3466
*B. lata* ATCC 17769	0.8581	1.5047	4.6846	2.3581
*B. multivorans* ATCC17616	0.0011	0.0012	26.1484	21.8644
*B. ubonensis* LMG 20358	0.0548	0.0867	0.6486	1.1232
*B. contaminans* LMG 16227	0.0183	0.0268	10.5396	9.1080
*B. dolosa* LMG 18943	0.1165	0.0839	18.6143	6.3400
*B. cepacia* LMG 1222	0.2369	0.4611	4.3806	5.0242
*B. vietnamiensis* LMG 22486	0.8721	1.1513	1.8217	1.8081
*B. pyrrocinia* LMG 14191	2.4287	3.4043	6.7670	6.6825

Mulcahy et al. [28] analyzed clonal pairs of early and late *P. aeruginosa* isolates from single CF patients and found that in the majority of these patients, cultures of late isolates contained increased numbers of drug-tolerant persister cells. We analyzed clonal pairs of early and late *Bcc* isolates, obtained from 14 CF patients. The MICs and the fraction of persister cells are shown in Table 2. In general, the MICs were very similar (i.e. within a two-fold dilution), indicating that the strains did not acquire resistance during the course of the infection. Only in four patients the persister fraction was higher in the late isolates. In three patients there was no difference between the early and the late isolates and in eight patients the number of surviving cells was lower in the late than in the early isolate. Our observations suggest that, unlike in *P. aeruginosa,* the fraction of persisters does not increase with the duration of colonisation.

### Induction of ROS by Tobramycin

**Figure 2:**
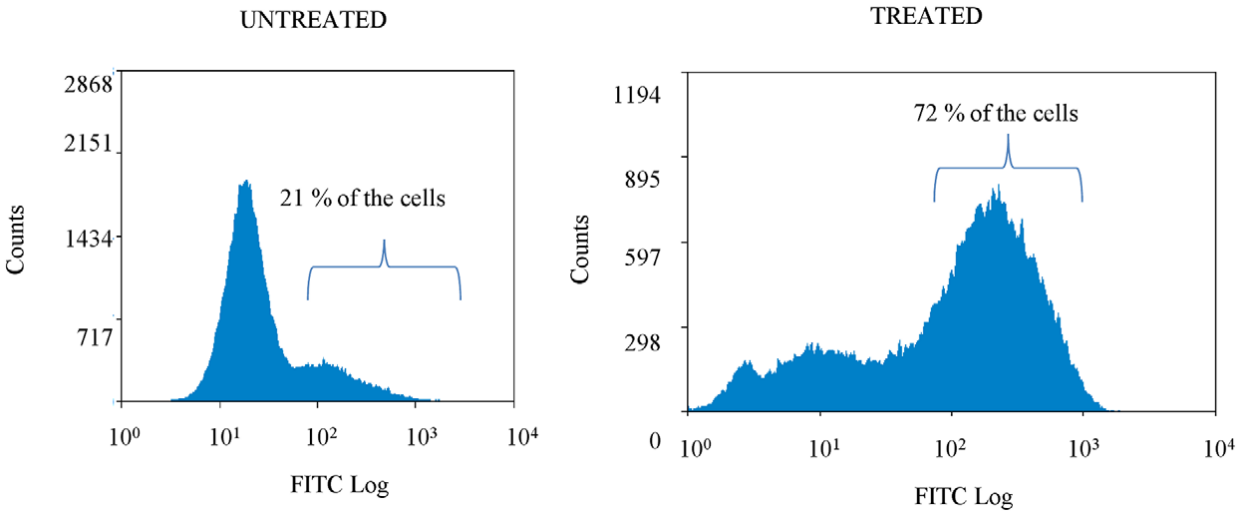
The number of cells in function of the DCHFDA-derived fluorescent signal for untreated and treated biofilms. Treated biofilms were exposed to tobramycin in a concentration of 4×MIC (1024 µg/ml) (24 h). The mean percentage of cells with a high fluorescent signal (>30) is indicated (n = 7). Differences between treated and untreated biofilms are statistically significant (p<0.05).

Recently, it was shown that bactericidal antibiotics induce the production of reactive oxygen species (ROS) [13]. Tobramycin is known to corrupt protein synthesis and by using ROS-specific staining and flow cytometry, we confirmed that tobramycin also drastically increased ROS production in treated sessile cells. Most of the cells in treated, unlike untreated biofilms showed a high fluorescent signal (Fig. 2).

### Gene Expression in Cells Treated with High Doses of Tobramycin

A transcriptome analysis showed a considerable change in gene expression in sessile cells treated with high doses of tobramycin relative to untreated sessile cells. Of the 8729* B. cenocepacia* J2315 sequences represented on the array, 2688 (30.8%) were significantly (p<0.05) upregulated, while the expression of 2413 sequences (27.6%) was significantly (p<0.05) downregulated. 669 (9.4%) protein-coding genes were significantly (p<0.05) upregulated by a factor of at least two, while the expression of 651 protein-coding genes (9.1%) was significantly (p<0.05) downregulated by a factor of at least two. In addition, a significant differential expression of two or more was noted for 270 intergenic regions (161 upregulated and 109 downregulated) and for 37 rRNA genes (all downregulated). Only a single tRNA gene was upregulated, but 31 tRNA genes were downregulated. A breakdown of differentially expressed protein-encoding genes by functional category is presented in supplementary data (Fig. S2) [29], [30].

In this study we focused on genes involved in the production of ROS and we found that the major pathways were all downregulated in cells surviving high doses of tobramycin treatment (Table 5). We observed that genes from the TCA cycle, including the majority of genes resulting in the production of NADH or FADH_2_, genes involved in the electron transport chain and the gene coding for ferredoxin reductase were downregulated in persister cells. On the other hand genes involved in production of NADPH, which plays a pivotal role in maintaining the proper cellular redox balance, were upregulated [31]. For example BCAL3276, the gene coding for NAD-kinase, an enzyme that mediates the formation of NADP (a key coenzym known to tilt cellular metabolism towards the synthesis of NADPH and away from the formation of NADH) was upregulated. Similarly, BCAL3395, the gene coding for malic enzyme, which is one of the NADPH producing enzymes, was also upregulated. Isocitrate dehydrogenase kinase-phosphatase, a bifunctional enzyme that can inactivate isocitrate dehydrogenase, thereby directing isocitrate to the glyoxylate shunt, as well as both isocitrate lyase (ICL) encodig genes were also found to be upregulated. In line with these findings, BCAL0813, encoding a RNA polymerase factor sigma 54 which represses the glyoxylate pathway [32] was downregulated. Glutamate dehydrogenase (BCAL3359) was upregulated and α-ketoglutarate dehydrogenase (BCAL1515) was downregulated indicating an increased production of α-ketoglutarate, a ROS scavenger. Other proteins involved in response to oxidative stress which were significantly upregulated include proteins involved in glutathione biosynthesis, thioredoxine, peroxiredoxine and glutathione peroxidase. The expression levels of selected genes were confirmed by qPCR (Table 5).

**Table 5:** Differences in gene expression expressed as fold changes in treated vs untreated biofilms.

Gene number	Annotation	Microarray	qPCR
**Glyoxylate shunt**			
BCAL2122	Malate synthase	1.4*	−3.3*
BCAL2118	Isocitrate lyase AceA	2.3*	1.9
BCAM1588	Isocitrate lyase	3.1*	1.6
BCAL0813	RNA polymerase factor sigma 54	−1.5*	−
BCAL1949	Glyoxylate carboligase	−1.5	−
**Tricarboxylic acid cycle**			
BCAM0961	Aconitate hydratase	−1.1	−
BCAM2701	Aconitate hydratase	−1.3*	−1.3
BCAM1833	Aconitate hydratase/methylisocitrate dehydratase	1.5*	1.1
BCAL2735	Isocitrate dehydrogenase	1.3*	−2.5*
BCAL2736	Isocitrate dehydrogenase	1.4	1.5
BCAL1515	α-ketoglutarate dehydrogenase E1	−2.0*	−
BCAL1516	Dihydrolipoamide succinyltransferase	−3.3*	−
BCAL1517	Dihydrolipoamide dehydrogenase	−2.5*	−
BCAL2207	Putative dihydrolipoamide dehydrogenase	−1.3*	−
BCAL1215	Dihydrolipoamide dehydrogenase	−1.4*	−
BCAL0956	Succinyl-CoA synthetase beta chain	−2.0*	−
BCAL0957	Succinyl-CoA synthetase subunit alpha	−3.3*	−10.0*
BCAM0967	Putative succinate dehydrogenase	−1.7*	−
BCAM0968	Putative succinate dehydrogenase	−2.5*	−
BCAM0969	Succinate dehydrogenase flavoprotein	−2.5*	−
BCAM0970	Succinate dehydrogenase iron-sulfur subunit	−5.0*	−25.0*
BCAL2908	Fumarate hydratase	−1.3*	1.9*
BCAL2287	Putative fumarate dehydrogenase	1.0	−
BCAM0965	Malate dehydrogenase	1.0	−2.0*
BCAL2746	Putative citrate synthase	−1.3	−
BCAM0964	Putative lyase	−1.4*	−
BCAS0023	HpcH/HpaI aldolase/citrate lyase family	−2.5*	−
BCAM0972	Type II citrate synthase	−5.0*	−
**Oxidative phosphorylation**			
BCAL2142	Cytochrome o ubiquinol oxidase subunit III	−2.0*	−
BCAL2143	Ubiquinol oxidase polypeptide I	−2.0*	−
BCAL0750	Cytochrome c oxidase polypeptide I	−1.7*	−
BCAL0752	Cytochrome c oxidase assembly protein	−2.5*	−
BCAL0753	Hypothetical protein	−2.5*	−
BCAL0754	Putative cytochrome c oxidase subunit III	−2.0*	−
BCAL0030–0037	F_0_F_1_ ATP synthase subunit A-ε	−1.7*–−2.5*°	−
BCAL2331–2343	NADH dehydrogenase subunit B-N	−2.5–−10.0*°	−
**NAD(P)H production**			
BCAL3276	NAD-kinase	1.4*	−
BCAL0672	Isocitrate dehydrogenase kinase/phosphatase	1.3*	−
BCAL3359	Glutamate dehydrogenase	4.2*	−
BCAL3395	Malic enzyme	1.7*	−
**Response to oxidative stress**			
BCAL1250	Putative glutathione S-transferase	1.6*	−
BCAL3331	Putative glutathione S-transferase	3.4*	−
BCAL0463	Putative thioredoxin	1.6*	−
BCAL2013	AhpC/TSA family protein	1.9*	−
BCAL2106	Glutathion peroxidase	1.6*	−
BCAM2318	Putative ferredoxin oxidoreductase	−10.0*	−33.3*
**Fe-storage**			
BCAM2627	Putative hemin ABC transporter protein	5.2*	−
BCAM2630	Hemin importer ATP binding subunit	2.8*	−
BCAM2224	Putative pyochelin receptor protein FptA	2.7*	−
BCAL1790	Putative iron-transport protein	2.5*	−
BCAL1347	Putative Fe uptake system extracellular binding protein	2.5*	−
BCAM2228	Putative pyochelin synthetase PchF	2.1*	−
BCAL1789	Putative iron-transport protein	2.0*	−
BCAL1371	Putative TonB-dependent siderophore receptor	2.0*	−
BCAL1702	Putative ornibactin biosynthesis protein	−2.2*	−

-: no qPCR experiments were performed. *: significant change in expression between the treated and the untreated biofilms (n = 3, p<0.05). °: range of fold changes for the various subunits.

### Eradication of Persister Cells

**Figure 3:**
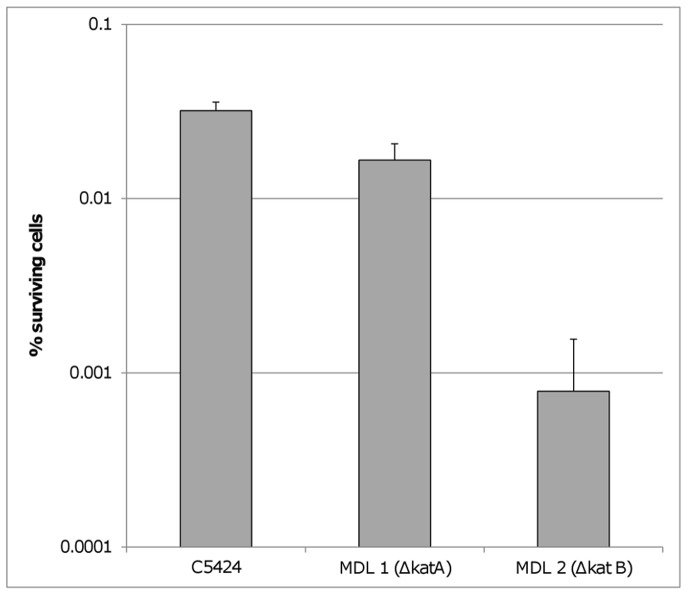
Effect of knocking out catalase function on survival of peristers. The number of surviving cells in biofilms formed by *B. cenocepacia* C5424 and two catalase mutants after treatment with tobramycin in a concentration of 4×MIC (512 µg/ml). Error bars represent standard deviation (n = 3).

**Figure 4:**
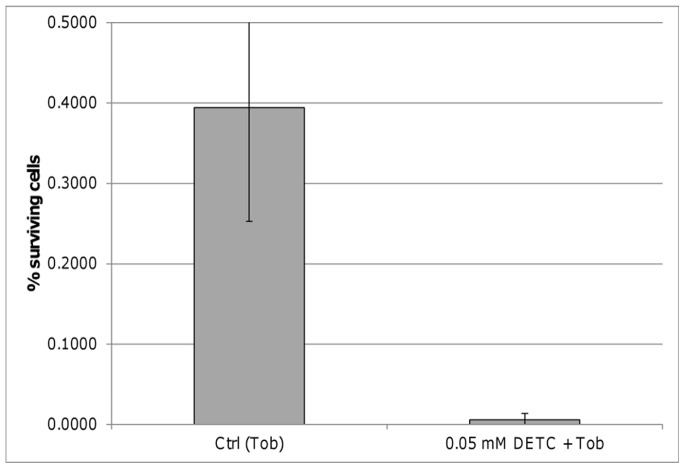
Effect of superoxide dismutase inhibition on survival of persisters. The number of surviving cells in *B. cenocepacia* J2315 biofilms after treatment with tobramycin alone (4×MIC, 24 h) or in combination with diethyldithiocarbamate (DETC 0.05 mM). Error bars represent standard deviation (n = 3, p<0.05).

Our transcriptome analysis suggested that protection against ROS is important in survival of persister cells. To confirm this we determined the number of persisters in biofilms formed by catalase mutants. *B. cenocepacia* contains two catalases of which one (KatA) is a specialized catalase/peroxidase which helps maintaining the normal activity of the TCA cycle, while KatB is a classical catalase/peroxidase which plays a global role in cellular protection against oxidative stress by converting toxic H_2_O_2_ into H_2_O and O_2_ [20]. We found the persister fraction to be slightly reduced in biofilms formed by the Δ*katA* mutant but almost 40-fold reduced in biofilms formed by the Δ*katB* mutant (Fig. 3). Furthermore, the number of surviving cells was almost 100 times lower after addition of the superoxide dismutase inhibitor diethyldithiocarbamate (DETC) (Fig. 4). Superoxide dismutases are antioxidant enzymes that detoxify O_2_^−.^ by a dismutation reaction generating H_2_O_2_ and O_2_ [16]_._ The inhibitor had no effect on untreated biofilms in the concentrations tested, but reduced the number of surviving cells in a concentration dependent manner when combined with tobramycin. Together, these results indicate that protection against ROS is indeed important for the survival of persister cells.

**Figure 5:**
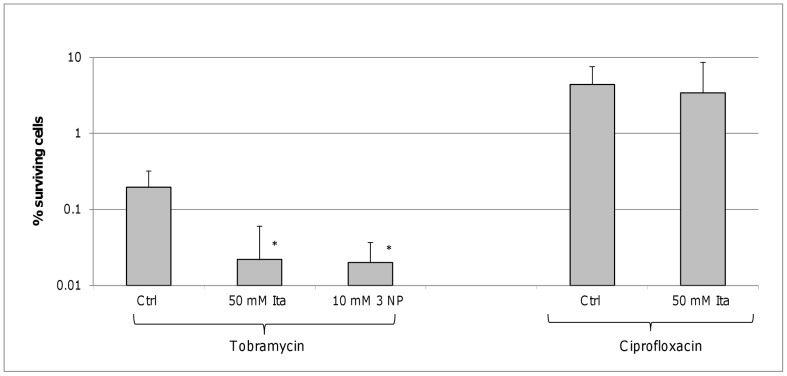
Effect of isocitrate lyase inhibition on *B. cenocepacia* J2315. The number of surviving cells in *B. cenocepacia* J2315 biofilms after treatment with tobramycin (4×MIC, 24 h) or ciprofloxacin (4×MIC, 24 h) for biofilms grown in LB or LB supplemented with 50 mM itaconate (ita) or 10 mM 3-nitropropionate (3-NP). Error bars represent standard deviation. Statistically significant differences are indicated by an asterix (p<0.05, n ≥3).

**Figure 6:**
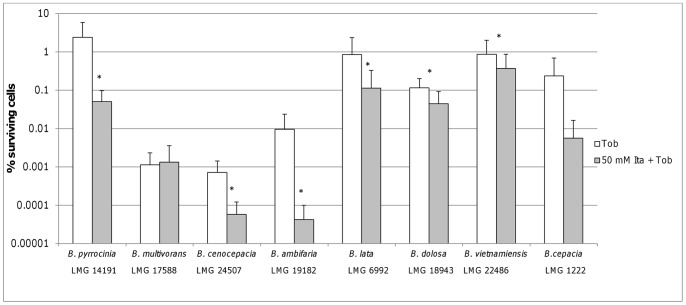
Effect of iocitrate lyase inhibition on different *Bcc* strains. The number of surviving cells in different *Bcc* biofilms after treatment with tobramycin (4×MIC, 24 h) for biofilms grown in LB or LB supplemented with 50 mM itaconate. Statistically significant differences are indicated by an asterix (p<0.05) (n≥3).

Persister cells are typically considered as “dormant” (i.e. metabolically inactive) cells [11] but our data challenge that paradigm. While it is true that some pathways (i.e. TCA cycle) were found to be downregulated, other pathways were upregulated, and these may be a novel therapeutic target for combination therapy. The importance of the upregulation of the glyoxylate shunt for the survival of persister cells was confirmed by inhibition of ICL, the first enzyme in the shunt. Because persisters are thought to be pre-existent, biofilms were grown in the presence of these inhibitors. During treatment the inhibitors were removed because of pH incompatibility with the antibiotics used. Pre-treatment of *B. cenocepacia* biofilms with the ICL inhibitor itaconate in a concentration of 50 mM reduced the persister fraction approximately 10 fold when the biofilms were subsequently treated with tobramycin. Pre-treatment with 10 mM 3-NP, a more potent ICL inhibitor resulted in similar reductions (Fig. 5). The concentrations of the ICL inhibitor used were below the MIC and they did not affect growth in the untreated biofilms. In addition, we did not observe an effect of either inhibitor on ROS production (data not shown). The additional killing observed after combined treatment with tobramycin and an ICL inhibitor was not observed with ciprofloxacin (Fig. 5). This may suggest that this effect is related to the type of antibiotic and/or the magnitude of its effect on biofilms. However, this remains to be investigated. Similar results were obtained in other *Bcc* bacteria (Fig. 6).

**Figure 7:**
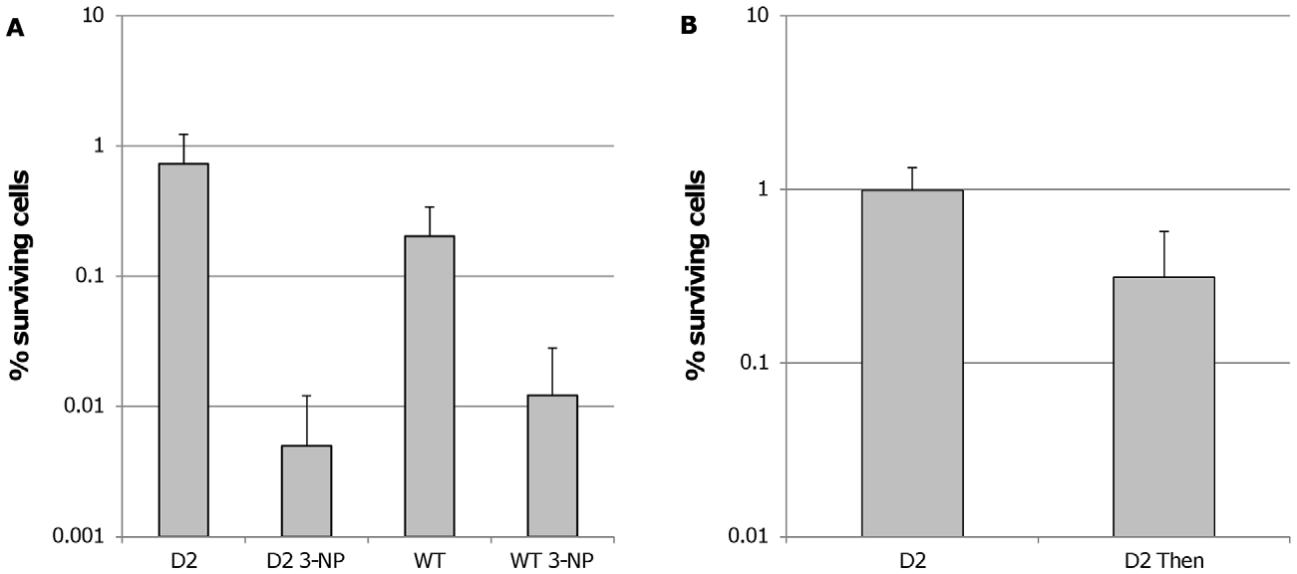
A. The number of surviving cells in a double ICL mutant (D2) compared to wild type (WT). The number of surviving cells in a double ICL mutant (D2) and in a wild type *B. cenocepacia* J2315 (WT) biofilm after treatment with tobramycin (4 MIC, 24h) for biofilms grown in LB or LB supplemented with 10 mM 3-NP. Error bars represent standard deviation (n ≥3). For both strains, treatment with 3-NP resulted in significantly (p<0.05) less surviving cells compared to the untreated control. B. Effect of succinate dehydrogenase inhibition on *B. cenocepacia* D2. The number of surviving cells in *B. cenocepacia* D2 biofilms after treatment with tobramycin (4×MIC, 24 h) for biofilms grown in LB or LB supplemented with 250 nM 2-thenoyltrifluoroacetone (Then). Error bars represent standard deviation (n = 4). Statistically significant differences are indicated by an asterix (p<0.05).

To confirm the results obtained by chemically inhibiting ICL, we constructed a *B. cenocepacia* J2315 mutant in which both ICL genes were inactivated. Surprisingly, there was no significant decrease in the number of surviving cells after treatment with tobramycin in the D2 mutant compared to wild type (Fig. 7A), but we did find an additional effect after adding 3-NP to the mutant. Itaconate and 3-NP are succinate analogues and besides inhibiting ICL, they also inhibit succinate dehydrogenase (SDH) [33].

We hypothesised that an upregulation of ICL in persisters not only limits the production of NADH (and thus limits ROS production) but also leads to an increased intracellular succinate concentration. Succinate can be oxidised by SDH, thereby generating FADH_2_ which can lead to basal production of ATP in persister cells in which other energy-generating systems are downregulated. Itaconate and 3-NP likely kill persisters by shutting down the remaining ATP production by inhibiting both ICL and SDH. To test this hypothesis we constructed a SDH antisense overexpression mutant in the D2 background, in which SDH is inactivated if rhamnose is present. However, we noticed that Tp (required to maintain the plasmid) and/or rhamnose (required to induce antisense RNA expression) had a mild influence of biofilm formation as such, and on the number of persisters in biofilms (data not shown), which made interpretation of these data impossible. However, addition of an ubiquinone type SDH inhibitor, 2-thenoyltrifluoroacetone, to *B. cenocepacia* D2 biofilms significantly (p<0.05) reduced the number of surviving persisters (Fig. 7B), while not affecting the MIC for tobramycin (data not shown).

The reaction catalysed by ICL also leads to the production of glyoxylate. In *B. cenocepacia* glyoxylate can be used to form malate (through the activity of malate synthase) or tartronate semialdehyde (through the activity of glyoxylate carboligase). There are two malate synthases of which only one (BCAL2122) was slightly upregulated and glyoxylate carboligase (BCAL1949) was slightly downregulated (Table 5).

### Conclusion

Our results contribute to a better understanding of the molecular mechanisms responsible for the antimicrobial tolerance of *Bcc* biofilms by demonstrating that these biofilms contain tolerant persister cells. In these surviving persister cells, the TCA cycle was downregulated and the expression of genes involved in the electron transport chain was also downregulated. This way the cells avoid the production of ROS. At the same time, persister cells activate an alternative pathway, i.e. the glyoxylate shunt. When biofilms were grown in the presence of an inhibitor of ICL and SDH, less persisters survived. Similar results were obtained in different species, indicating that this mechanism is widespread within the *Bcc*. So far, most anti-persister strategies focussed on “reawaking” persisters for efficient killing [34], but we found that inhibiting a cellular target can also reduce the number of surviving persisters. This finding may provide novel approaches for treatment of persister-related infections.

## Supporting Information

Figure S1 **Average expression stability values (M) of remaining control genes during stepwise exclusion of the least stable control gene (between brackets).** Genes are ranked from left to right in order of increasing expression stability (decreasing M value). Genes labeled with an asterix were used for normalization.
(TIF)

Figure S2 **Differentially expressed protein-coding genes ordered by functional category.**
(DOCX)
