# Supplementary figures and images for: Biofilm-Grown Burkholderia cepacia Complex Cells Survive Antibiotic Treatment by Avoiding Production of Reactive Oxygen Species
Source: PLoS One. 2013 Mar 13;8(3):e58943. doi: 10.1371/journal.pone.0058943 (PMC3596321; doi:10.1371/journal.pone.0058943)

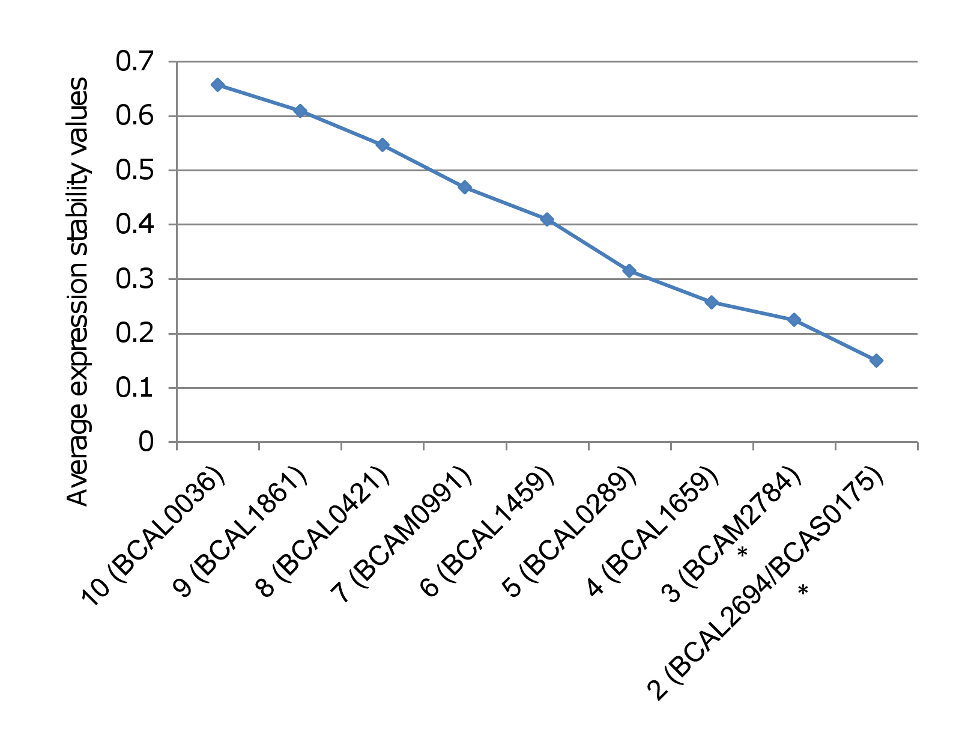

Supplement: Figure S1 — Average expression stability values (M) of remaining control genes during stepwise exclusion of the least stable control gene (between brackets). Genes are ranked from left to right in order of increasing expression stability (decreasing M value). Genes labeled with an asterix were used for normalization. (TIF) [file pone.0058943.s001.tif]
