# Supplementary material for: Biofilm-Grown Burkholderia cepacia Complex Cells Survive Antibiotic Treatment by Avoiding Production of Reactive Oxygen Species
Source: PLoS One. 2013 Mar 13;8(3):e58943. doi: 10.1371/journal.pone.0058943 (PMC3596321; doi:10.1371/journal.pone.0058943)
Supplement: Figure S2 — Differentially expressed protein-coding genes ordered by functional category. (DOCX) [file pone.0058943.s002.docx]

Figure S 2 : Differentially expressed protein-coding genes ordered by functional category [29, 30]

| **Functional category** | **Code** | **Probes** | **Up** | **Down** | **% up** | **% down** |
| --- | --- | --- | --- | --- | --- | --- |
| Hypothetical protein | 0.0.0 | 145 | 16 | 19 | 11% | 13% |
| Conserved hypothetical protein | 0.0.2 | 871 | 108 | 82 | 12% | 9% |
| Chemotaxis and mobility | 1.1.1 | 72 | 3 | 0 | 4% | 0% |
| Chromosome replication | 1.2.1 | 11 | 4 | 1 | 36% | 9% |
| Chaperones | 1.3.1 | 24 | 3 | 3 | 13% | 13% |
| **Protection responses** |  |  |  |  |  |  |
| Cell killing | 1.4.1. | 5 | 2 | 0 | 40% | 0% |
| Detoxification | 1.4.2 | 42 | 3 | 1 | 7% | 2% |
| Drug-analog sensitivity | 1.4.3 | 89 | 6 | 4 | 7% | 5% |
| **Transport/ binding proteins** |  |  |  |  |  |  |
| Transport/binding proteins | 1.5.0 | 478 | 35 | 19 | 7% | 4% |
| Amino acids and amines | 1.5.1 | 101 | 12 | 7 | 12% | 7% |
| Cations | 1.5.2 | 44 | 9 | 6 | 21% | 14% |
| Carbohydrates, organic acids and alcohols | 1.5.3 | 63 | 3 | 7 | 5% | 11% |
| Anions | 1.5.4 | 15 | 2 | 1 | 13% | 7% |
| Other | 1.5.5 | 97 | 4 | 6 | 4% | 6% |
| **Adaptation** |  |  |  |  |  |  |
| Adaptation, atypical conditions | 1.6.1 | 32 | 4 | 6 | 13% | 19% |
| Osmotic adaptation | 1.6.2 | 11 | 1 | 3 | 9% | 27% |
| Fe storage | 1.6.3 | 38 | 7 | 1 | 18% | 3% |
| **Cell division** |  |  |  |  |  |  |
| Cell division | 1.7.1 | 23 | 2 | 2 | 9% | 9% |
| **Macromolecule metabolism** |  |  |  |  |  |  |
| Macromolecule degradation | 2.1.0 | 1 | 0 | 0 | 0% | 0% |
| Degradation of DNA | 2.1.1 | 9 | 0 | 0 | 0% | 0% |
| Degradation of RNA | 2.1.2 | 11 | 1 | 0 | 9% | 0% |
| Degradation of polysaccharides | 2.1.3 | 8 | 0 | 1 | 0% | 13% |
| Degradation of proteins, peptides glycoproteins | 2.1.4 | 38 | 2 | 2 | 5% | 5% |
| **Macromolecules synthesis- modification** |  |  |  |  |  |  |
| Macromolecules synthesis-modification | 2.2.0 | 3 | 1 | 3 | 33% | 27% |
| Amino acyl tRNA synthesis, tRNA modification | 2.2.1 | 42 | 0 | 8 | 0% | 19% |
| Basic protein- synthesis, modification | 2.2.2 | 5 | 0 | 0 | 0% | 0% |
| DNA-replication, repair, restriction, modification | 2.2.3 | 67 | 7 | 12 | 11% | 18% |
| Lipopolysaccharide | 2.2.5 | 55 | 4 | 12 | 7% | 22% |
| Lipoprotein | 2.2.6 | 3 | 0 | 1 | 0% | 33% |
| Phospholipids | 2.2.7 | 9 | 0 | 0 | 0% | 0% |
| Polysaccharides | 2.2.8 | 3 | 0 | 2 | 0% | 67% |
| Protein modification | 2.2.9 | 20 | 0 | 2 | 0% | 10% |
| Proteins - translation and modification | 2.2.10 | 28 | 0 | 5 | 0% | 18% |
| RNA synthesis, modification, DNA transcription | 2.2.11 | 36 | 1 | 7 | 3% | 19% |
| tRNA | 2.2.12 | 47 | 0 | 0 | 0% | 0% |
| **Metabolism of small molecules** |  |  |  |  |  |  |
| Metabolism of small molecules | 3.0.0 | 2 | 1 | 0 | 50.% | 0% |
| Amino acid biosynthesis | 3.1.0 | 9 | 1 | 1 | 11% | 11% |
| Arginine | 3.1.2 | 9 | 0 | 2 | 0% | 22% |
| Asparagine | 3.1.3 | 1 | 0 | 1 | 0% | 100% |
| Aspartate | 3.1.4 | 1 | 0 | 0 | 0% | 0% |
| Chorismate | 3.1.5 | 1 | 0 | 0 | 0% | 0% |
| Cysteine | 3.1.6 | 4 | 0 | 0 | 0% | 0% |
| Glutamate | 3.1.7 | 3 | 1 | 0 | 33% | 0% |
| Glutamine | 3.1.8 | 3 | 0 | 1 | 0% | 33% |
| Glycine | 3.1.9 | 5 | 0 | 0 | 0% | 0% |
| Histidine | 3.1.10 | 11 | 0 | 0 | 0% | 0% |
| Isoleucine | 3.1.11 | 3 | 0 | 0 | 0% | 0% |
| Leucine | 3.1.12 | 7 | 2 | 0 | 29% | 0% |
| Lysine | 3.1.13 | 7 | 0 | 0 | 0% | 0% |
| Methionine | 3.1.14 | 10 | 0 | 1 | 0% | 10% |
| Phenylalanine | 3.1.15 | 2 | 2 | 0 | 100% | 0% |
| Proline | 3.1.16 | 3 | 0 | 0 | 0% | 0% |
| Serine | 3.1.17 | 2 | 0 | 1 | 0% | 50% |
| Threonine | 3.1.18 | 3 | 0 | 2 | 0% | 67% |
| Tryptophan | 3.1.19 | 11 | 0 | 1 | 0% | 9% |
| Tyrosine | 3.1.20 | 1 | 0 | 1 | 0% | 100% |
| Valine | 3.1.21 | 3 | 0 | 2 | 0% | 67% |
| **Biosynthesis of cofactors, carriers** |  |  |  |  |  |  |
| Biosynthesis of cofactors, carriers | 3.2.0 | 5 | 0 | 1 | 0% | 20% |
| ACP | 3.2.1 | 9 | 4 | 0 | 44% | 0% |
| Biotin | 3.2.2 | 5 | 0 | 0 | 0% | 0% |
| Cobalamin | 3.2.3 | 13 | 2 | 0 | 15% | 0% |
| Folic acid | 3.2.5 | 10 | 0 | 04 | 0% | 40% |
| Heme, porphyrin | 3.2.6 | 26 | 0 | 0 | 0% | 0% |
| Lipoate | 3.2.7 | 3 | 0 | 0 | 0% | 0% |
| Menaquinone, ubiquinone | 3.2.8 | 10 | 1 | 4 | 10% | 40% |
| Molybdopterin | 3.2.9 | 10 | 0 | 0 | 0% | 0% |
| Pantothenate | 3.2.10 | 8 | 0 | 1 | 0% | 13% |
| Pyridine nucleotide | 3.2.11 | 6 | 1 | 1 | 17% | 17% |
| Pyridoxine | 3.2.12 | 8 | 1 | 1 | 13% | 13% |
| Riboflavin | 3.2.13 | 9 | 1 | 2 | 11% | 22% |
| Thiamin | 3.2.14 | 10 | 0 | 1 | 0% | 10% |
| Thioredoxin, glutaredoxin, glutathione | 3.2.15 | 11 | 1 | 2 | 9% | 18% |
| BCCP | 3.2.16 | 3 | 0 | 0 | 0% | 0% |
| **Central intermediary metabolism** |  |  |  |  |  |  |
| Entner-Douderoff | 3.3.3 | 2 | 0 | 0 | 0% | 0% |
| Gluconeogenesis | 3.3.4 | 7 | 0 | 2 | 0% | 29% |
| Glyoxylate | 3.3.5 | 13 | 1 | 1 | 8% | 8% |
| Misc. glucose metabolism | 3.3.7 | 1 | 1 | 0 | 100% | 0% |
| Misc. glycerol metabolism | 3.3.8 | 1 | 1 | 0 | 100% | 0% |
| Non-oxidative branch, pentose pathway | 3.3.9 | 4 | 0 | 0 | 0% | 0% |
| Nucleotide hydrolysis | 3.3.10 | 4 | 0 | 0 | 0% | 0% |
| Nucleotide interconversions | 3.3.11 | 13 | 0 | 1 | 0% | 8% |
| Phosphorus compounds | 3.3.13 | 7 | 0 | 1 | 0% | 14% |
| Polyamine biosynthesis | 3.3.14 | 14 | 2 | 1 | 14% | 7% |
| Pool, multipurpose conversions of intermed. metabolism | 3.3.15 | 72 | 7 | 8 | 10% | 11% |
| S-adenosyl methionine | 3.3.16 | 1 | 0 | 0 | 0% | 0% |
| Salvage of nucleosides and nucleotides | 3.3.17 | 13 | 1 | 1 | 8% | 8% |
| Sugar-nucleotide biosynthesis, conversions | 3.3.18 | 3 | 0 | 1 | 0% | 33% |
| Sulfur metabolism | 3.3.19 | 18 | 4 | 0 | 22% | 0% |
| Amino acids | 3.3.20 | 15 | 0 | 0 | 0% | 0% |
| Amino acids: other | 3.3.21 | 36 | 0 | 6 | 0% | 17% |
| **Degradation** |  |  |  |  |  |  |
| Degradation | 3.4.0 | 19 | 3 | 0 | 16% | 0% |
| Amines | 3.4.1 | 22 | 1 | 2 | 5% | 9% |
| Amino acids | 3.4.2 | 58 | 7 | 5 | 12% | 9% |
| Carbon compounds | 3.4.3 | 117 | 5 | 13 | 4% | 11% |
| Fatty acids | 3.4.4 | 34 | 6 | 3 | 18% | 9% |
| Other | 3.4.5 | 75 | 5 | 6 | 7% | 8% |
| ATP-proton motive force | 3.4.6 | 0 | 0 | 0 |  |  |
| **Energy metabolism, carbon** |  |  |  |  |  |  |
| Aerobic respiration | 3.5.1 | 28 | 3 | 15 | 11% | 54% |
| Anaerobic respiration | 3.5.2 | 10 | 0 | 2 | 0% | 20% |
| Electron transport | 3.5.3 | 72 | 3 | 10 | 4% | 14% |
| Fermentation | 3.5.4 | 3 | 0 | 0 | 0% | 0% |
| Glycolysis | 3.5.5 | 12 | 0 | 1 | 0% | 8% |
| Oxidative branch, pentose pathway | 3.5.6 | 4 | 0 | 0 | 0% | 0% |
| Pyruvate dehydrogenase | 3.5.7 | 2 | 0 | 0 | 0% | 0% |
| TCA cycle | 3.5.8 | 22 | 0 | 9 | 0% | 41% |
| ATP | 3.5.9 | 12 | 0 | 5 | 0% | 42% |
| **Fatty acid biosynthesis** |  |  |  |  |  |  |
| Fatty acid and phoshatic acid biosythesis | 3.6.1 | 22 | 4 | 1 | 18% | 5% |
| **Nucleotide biosynthesis** |  |  |  |  |  |  |
| Purine ribonucleotide biosynthesis | 3.7.1 | 16 | 0 | 2 | 0% | 13% |
| Pyrimidine ribonucleotide biosynthesis | 3.7.2 | 12 | 0 | 2 | 0% | 17% |
| **Cell envelop** |  |  |  |  |  |  |
| Periplasmatic/exported/lipoproteins | 4.1.0 | 465 | 57 | 38 | 12% | 8% |
| Inner membrane | 4.1.1 | 392 | 29 | 32 | 7% | 8% |
| Murein sacculus, peptidoglycan | 4.1.2 | 38 | 5 | 6 | 13% | 16% |
| Outer membrane constituents | 4.1.3 | 63 | 8 | 3 | 13% | 5% |
| Surface polysaccharides and antigens | 4.1.4 | 66 | 1 | 20 | 2% | 30% |
| Surface structures | 4.1.5 | 38 | 1 | 3 | 3% | 8% |
| **Ribosome constituents** |  |  |  |  |  |  |
| Ribosomal and stable RNAs | 4.2.1 | 1 | 0 | 0 | 0% | 0% |
| Ribosomal proteins-synthesis, modification | 4.2.2 | 57 | 0 | 34 | 0% | 60% |
| Ribosomes-maturation and modification | 4.2.3 | 4 | 0 | 3 | 0% | 75% |
| **Extrachromosomal** |  |  |  |  |  |  |
| Colicin-related functions | 5.1.1 | 2 | 0 | 1 | 0% | 50% |
| Phage-related functions and prophages | 5.1.2 | 276 | 38 | 8 | 14% | 3% |
| Plasmid-related functions | 5.1.3 | 26 | 3 | 2 | 12% | 8% |
| Transposon-related functions | 5.1.4 | 71 | 6 | 1 | 9% | 1% |
| Rest | 5.1.5 | 33 | 2 | 3 | 6% | 9% |
| **Global functions** |  |  |  |  |  |  |
| Global functions | 6.0.0 | 14 | 3 | 1 | 21% | 7% |
| Global regulatory functions | 6.1-6.5 | 768 | 88 | 39 | 11% | 5% |
| **Not classified** | 7.0.0 | 1281 | 112 | 105 | 9% | 8% |
